# C_60_ Fullerene Reduces the Development of Post-Traumatic Dysfunction in Rat Soleus Muscle

**DOI:** 10.3390/ijms252212206

**Published:** 2024-11-14

**Authors:** Yuriy Prylutskyy, Dmytro Nozdrenko, Olexandr Motuziuk, Svitlana Prylutska, Kateryna Bogutska, Olga Abramchuk, Alevtyna Morenko, Daria Franskevych, Peter Scharff, Uwe Ritter

**Affiliations:** 1 ESC “Institute of Biology and Medicine”, Taras Shevchenko National University of Kyiv, 01601 Kyiv, Ukraine; prylut@ukr.net (Y.P.); dmytro.nozdrenko@knu.ua (D.N.); motuziuk.oleksandr@vnu.edu.ua (O.M.); bogutska_ki@knu.ua (K.B.); dashaqq@gmail.com (D.F.); 2 Faculty of Biology and Forestry, Lesya Ukrainka Volyn National University, 43025 Lutsk, Ukraine; abramchuk.olga@vnu.edu.ua (O.A.); morenko.alevtyna@vnu.edu.ua (A.M.); 3 Faculty of Plant Protection, Biotechnology and Ecology, National University of Life and Environmental Science of Ukraine, 03041 Kyiv, Ukraine; psvit_1977@ukr.net; 4 Institute of Chemistry and Biotechnology, Technical University of Ilmenau, 98693 Ilmenau, Germany; peter.scharff@tu-ilmenau.de

**Keywords:** soleus muscle, muscle injury, C_60_ fullerene, biomechanical and biochemical parameters

## Abstract

Traumatic skeletal muscle injury is a complex pathology caused by high-energy trauma to muscle tissue. Previously, a positive effect was established when C_60_ fullerene was administered against the background of muscle ischemia, mechanical muscle injury, and other muscle dysfunctions, which probably protected the muscle tissue from damage caused by oxidative stress. Using tensiometry and biochemical analysis, the biomechanical parameters of skeletal muscle contraction and biochemical indices of the blood of rats 15 days after traumatic injury of the soleus muscle caused by myocyte destruction by compression were studied. The intraperitoneal administration of C_60_ fullerene aqueous solution (C_60_FAS) in a daily dose of 1 mg/kg improved its contractile function by 28–40 ± 2% and the values of the investigated biochemical indices of the animals’ blood by 15–34 ± 2% relative to the trauma group. The obtained results indicate the potential ability of C_60_ fullerenes, as powerful antioxidants, to reduce the development of post-traumatic dysfunction of the soleus muscle.

## 1. Introduction

A traumatic skeletal muscle injury is a complex pathology caused by high-energy trauma to muscle tissue. Approximately 70% of combat injuries are associated with skeletal muscle injury [1]. Muscle injuries are also a common part of sports pathologies [2]. One limitation of effective therapy for muscle injuries is the lack of a unified approach to classifying muscle injuries [3]. One of the types of mechanical muscle damage is a compression injury. Such injuries are often observed following man-made disasters, road traffic accidents, and military operations [4]. The rapid death observed in some cases is due to cardiac arrhythmia caused by high concentrations of intracellular electrolytes, such as potassium, suddenly washing over the heart. Further complications are mainly due to the development of renal failure. For the effective treatment of the damaged muscular system, the timing of the start of resuscitation procedures is critical: cell death as a result of crushing occurs within 1 h after the injury, which leads to necrosis and the subsequent destruction of the muscle, causing the release of intracellular contents into the plasma [5]. Common consequences of skeletal muscle crush syndrome include hypovolemic shock, hyperkalemia, hyperphosphatemia, hypocalcemia, metabolic acidosis, arrhythmia, and disseminated intravascular coagulation [6]. After an injury, muscle fibers can repair local damage, but they must be fully regenerated following myofibril necrosis [7]. It has been found that several weeks of non-invasive and drug-free muscle recovery after a severe injury does not lead to significant muscle regeneration, and this process is characterized by persistent inflammation, chronic activation of profibrotic markers, and extracellular matrix [8,9]. Infiltrating inflammatory and resident stem cells are involved before the repair of damaged muscle tissue. At the same time, during an intense inflammatory process, fibroblasts remain active, whereas the reparative capacity of stem cells decreases [10]. The excessive concentration of free radicals due to the inflammatory process has a detrimental effect on the regeneration processes [11]. Therefore, anti-inflammatory agents are widely used to treat muscle injuries [12], although they often lose their effectiveness due to numerous side effects [13]. For example, the immunosuppressive drug FK506 has improved the recovery of maximal muscle strength in the early phase of posttraumatic regeneration [14]. Using the antihypertensive drug Losartan reduced fibrosis and improved the regeneration of skeletal muscle injuries [15]. However, no improvement in muscle regeneration was observed in severe injuries, and the drug even negatively affected muscle function. In the absence of sufficient regeneration, the increased amount of connective tissue impairs the ability of the injured muscle to generate force, leading to decreased muscle function and impaired correction of accurate positioning. A positive therapeutic outcome has been achieved by using drugs that reduce inflammation and thereby regulate post-traumatic muscle fibrosis, the excess of which increases muscle stiffness and the risk of re-injury [16].

Due to their nanosized (almost spherical) shape, hydrophobicity, and unique chemical structure (the presence of double electron-deficient bonds), C_60_ fullerenes penetrate cells and localize preferentially in mitochondria [17], and they exhibit a strong reducing ability and act within in vitro and in vivo systems as powerful scavengers of free radicals [18,19], the overproduction of which leads to many pathologies. This opens up a valuable opportunity for the use of these nanoantioxidants, with effects surpassing those of well-known natural antioxidants—vitamins C, E, and carotenoids [20]. In vivo models previously demonstrated that the antioxidant properties of biocompatible C_60_ fullerenes [21] increased the force response of skeletal muscles after glyphosate poisoning [22] and muscle atrophy caused by prolonged immobilization [23]. A preliminary positive effect of C_60_ fullerene on soleus muscle contraction dynamics in rats after initiating ischemic injury [24] and mechanical trauma [25] was demonstrated. Thus, it can be assumed that the powerful antioxidant properties of C_60_ fullerene [18,19,20], reducing the level of the inflammatory process in the injured muscle of varying severity, will influence the efficiency of its post-traumatic recovery, which was the aim of the current study within the framework of the analysis of the biomechanical parameters of soleus muscle contraction and blood biochemical indices of model animals.

## 2. Results and Discussion

### 2.1. Biomechanical Analysis

**Figure 1:**
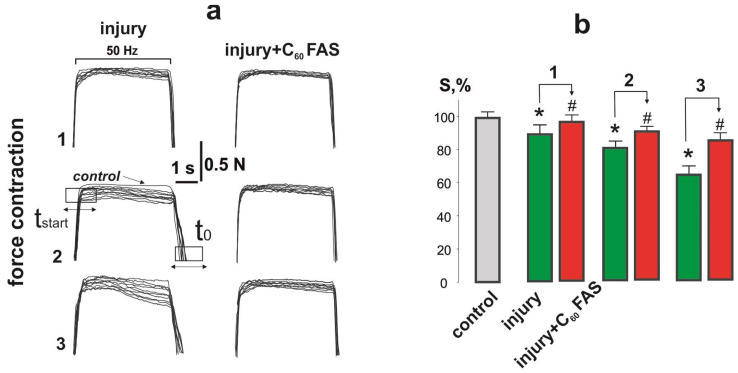
Force generation curves of soleus muscle contraction 15 days after its injury: mechanograms of muscle contraction (**a**); integrated muscle power (S; relative to the control, which was taken as 100%) (**b**); injury and injury + C_60_FAS—injury group (*n* = 30) and injury group against the background of C_60_FAS injection (*n* = 30), respectively; 1, 2, and 3—the severity of muscle injury; t_start_—the time between the beginning of stimulation and the beginning of muscle contraction; t_0_—the time between the end of stimulation and the power curve reaching the initial level; * *p* < 0.05 relative to the control group (*n* = 30); # *p* < 0.05 relative to the injury group (*n* = 30).

Figure 1 shows the mechanograms of 10 consecutive contractions of the soleus muscle obtained on the 15th day after the initiation of muscle injury of different degrees of severity at 5 s of non-relaxation stimulation at a frequency of 50 Hz.

The integrated muscle power decreased by 11 ± 1%, 19 ± 1%, and 34 ± 2% at the 1st, 2nd, and 3rd degrees of injury severity, respectively, compared to the control group, indicating incomplete regeneration of damaged muscle fibers during this period at the 2nd and 3rd degrees of injury severity. The application of C_60_ fullerene aqueous solution (C_60_FAS) injections increased the level of integrated muscle power: it almost normalized at the first injury severity grade and differed from the control group by 10 ± 1% and 14 ± 1% at the 2nd and 3rd injury severity grades, respectively (Figure 1).

The change in the value of the minimum force of muscle contraction was the main indicator of its dysfunction in each successive contractile act [26].

**Figure 2:**
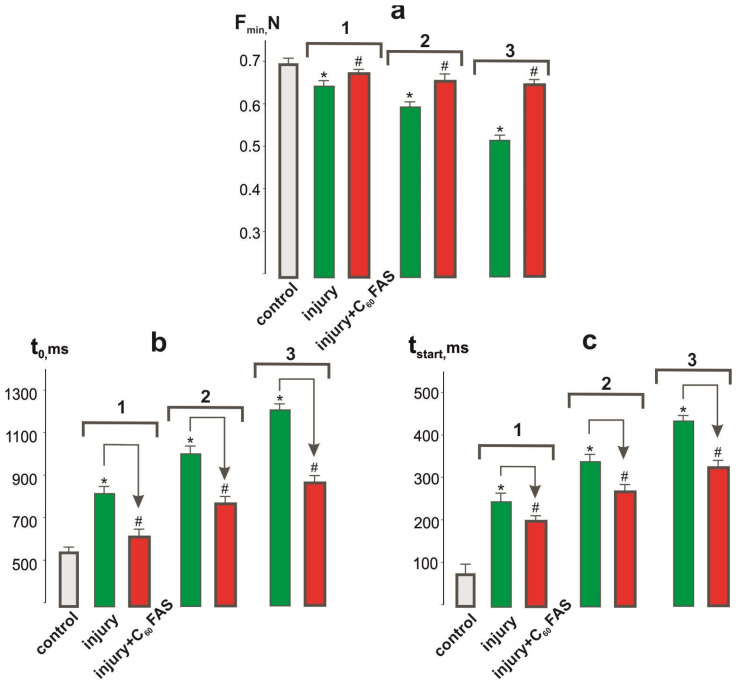
The biomechanical parameters of soleus muscle contraction 15 days after its injury: F_min_—minimal force of muscle contraction (**a**); t_0_—time between the end of stimulation and the force curve reaching the initial level (**b**); t_start_—time between the beginning of stimulation and the beginning of muscle contraction (**c**); injury and injury + C_60_FAS—injury group (*n* = 30) and injury group against the background of C_60_FAS injections (*n* = 30), respectively; 1, 2, and 3—severity of muscle injury; * *p* < 0.05 relative to the control group (*n* = 30); # *p* < 0.05 relative to the injury group (*n* = 30).

The level of minimal force was 0.64 ± 0.05, 0.59 ± 0.05, and 0.53 ± 0.05 N at the 1st, 2nd, and 3rd degrees of injury severity, respectively, and in the control group—0.70 ± 0.05 N. At the C_60_FAS injections, the value of this parameter was almost normalized and was 0.68 ± 0.05 N for all degrees of injury severity (Figure 2a).

One of the main factors influencing the decrease in the biomechanical parameters described above was the increase in the stiffness components of the muscle during post-traumatic regeneration [27].

To analyze them, we measured the time between the end of the stimulation and the exit of the force curve to the initial level (Figure 2b). The t_0_ time value increased from 560 ± 12 ms in the control group to 830 ± 14, 1050 ± 9, and 1190 ± 11 ms in injury severity 1, 2, and 3, respectively. The application of C_60_FAS reduced this parameter to 580 ± 10, 730 ± 8, and 850 ± 11 ms at the 1st, 2nd, and 3rd injury severity grades, respectively. Thus, the efficacy of the C_60_FAS therapy was 68–71 ± 4%.

The formation of muscle dysfunctions is also affected by changes in nerve conduction, the disruption of which is associated with the development of the inflammatory process occurring in the post-traumatic period [28]. The change in the onset of the force response of a muscle caused by a single stimulation pool allows us to assess the level of pathological changes in the neuromuscular preparation during prolonged, static, and slow dynamic reactions of the muscular system.

The value of the t_start_ time (Figure 2c) increased from 85 ± 2 ms in the control group to 270 ± 6, 345 ± 7, and 430 ± 11 ms in injury severity groups 1, 2, and 3, respectively. When C_60_FAS was applied, these values were 185 ± 12, 281 ± 8, and 315 ± 7 ms at the 1st, 2nd, and 3rd injury severity levels, respectively. Thus, the effect of C_60_FAS was 21 ± 1%, 18 ± 1%, and 26 ± 1% at the 1st, 2nd, and 3rd injury severity levels, respectively, relative to the trauma group.

A post-traumatic increase in muscle stiffness causes increased fatigue processes in active muscles [29].

**Figure 3:**
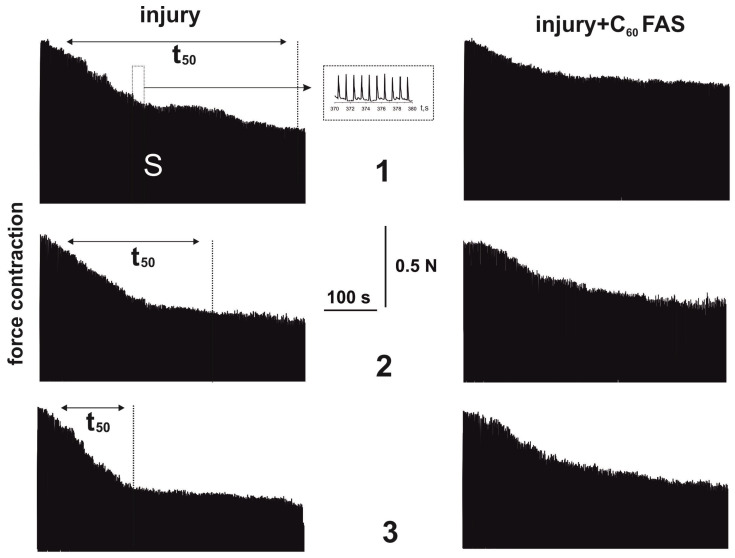
The mechanograms of soleus muscle contraction induced by the stimulation frequency of 1 Hz with a duration of 500 s, on the 15th day after traumatic injury initiation: injury and injury + C_60_FAS—injury group (*n* = 30) and injury group against the background of C_60_FAS injection (*n* = 30), respectively; 1, 2, and 3—severity of muscle injury; t_50_—time of contraction force reduction by 50% from the initial level; S—integrated muscle power.

**Figure 4:**
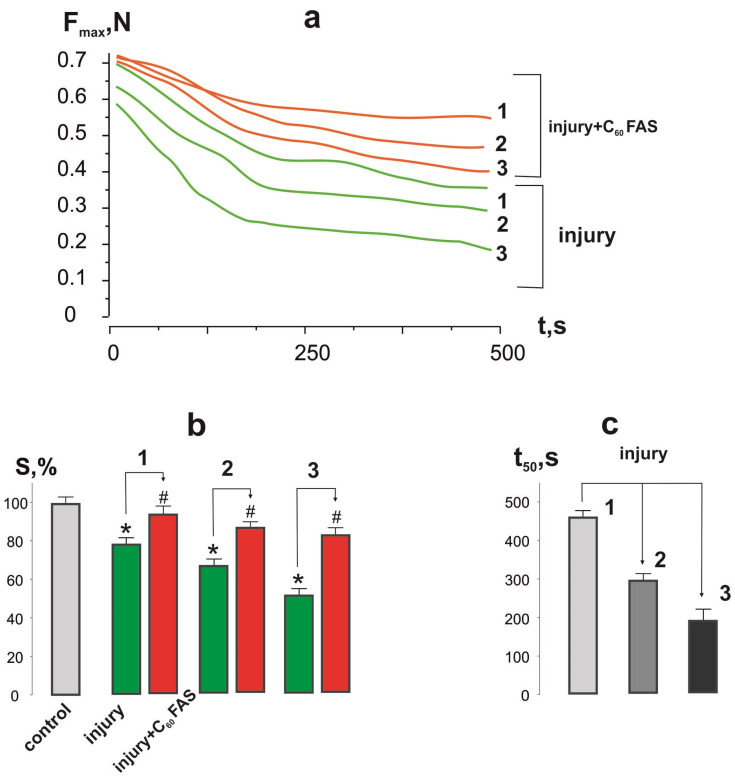
The biomechanical parameters of soleus muscle contraction 15 days after its injury: curves of changes in maximum contraction forces (F_max_) during 500 s of stimulation pool (**a**); changes in integrated muscle power (S) during 500 s of stimulation (**b**); time of reduction of contraction force by 50% from the initial level (t_50_) (**c**); injury and injury + C_60_FAS—injury group (*n* = 30) and injury group against the background of C_60_FAS injections (*n* = 30), respectively; 1, 2, and 3—severity of muscle injury; * *p* < 0.05 relative to the control group (*n* = 30); # *p* < 0.05 relative to the injury group (*n* = 30).

The development of muscle fatigue was assessed by calculating the time to reach 50% of the force response level when 1 Hz non-relaxation stimulation was applied (Figure 3). It should be noted that the value of this parameter in the control group was characterized by a long time interval. When registering force responses to the stimulation signal, a decrease in the force activity of the injured muscle was revealed (Figure 3)—in particular, the maximal force from 21 ± 1% at the 1st degree of injury severity to 64 ± 3% at the 3rd degree of injury severity relative to the control group (Figure 4a). The administration of C_60_FAS increased this index by 25 ± 1%, 34 ± 2%, and 53 ± 3% at injury severity grades 1, 2, and 3, respectively, relative to the injury group.

The decrease in integrated muscle power (Figure 4b) was 21 ± 1%, 33 ± 2%, and 49 ± 2% at the 1st, 2nd, and 3rd injury severity grades, respectively, relative to the control group. C_60_FAS injections increased this index by 18 ± 1%, 23 ± 1%, and 38 ± 2% at the 1st, 2nd, and 3rd injury severity levels, respectively, providing direct evidence of its positive effect on reducing connective and fibrotic tissue formation in the post-traumatic period.

The 1st, 2nd, and 3rd injury severity levels, respectively, achieved a 50% force response from the initial level, with values of 480 ± 5, 303 ± 2, and 202 ± 3 s (Figure 4c). The use of C_60_FAS injections stopped the progressive fatigue processes in the injured muscle.

Summarizing the above biomechanical results, we can conclude that the administration of C_60_FAS in a daily dose of 1 mg/kg into the injured soleus muscle during the experiment improved its contractile function by 28–40 ± 2% relative to the trauma group. This can be explained by the powerful antioxidant properties of C_60_ fullerenes [18,19,20], and this conclusion is in accord with our previous results [22,23,24,25], although it requires confirmation at the biochemical level.

### 2.2. Biochemical Analysis

The increased stiffness components of post-traumatic muscle tissue and the insufficient regeneration of damaged muscle fibers lead to high energy expenditure during the muscle’s functioning. The analysis of blood biochemical markers, in particular, creatinine and lactate levels, as well as creatine phosphokinase (CPK) and lactate dehydrogenase (LDH) activities, allows us to evaluate the physiological changes in the muscle and the therapeutic effect of the applied drug [30]. Studies have shown that all of these biochemical markers have a pronounced tendency to increase with the increasing severity of the injury, indicating that the muscle system performs work that is super-intensive for its physiological level and subsequently develops muscle fatigue.

Changes in creatinine concentration due to the destruction of intramuscular structures allow us to assess the residual level of myocyte damage and the efficiency of the post-traumatic repair.

**Figure 5:**
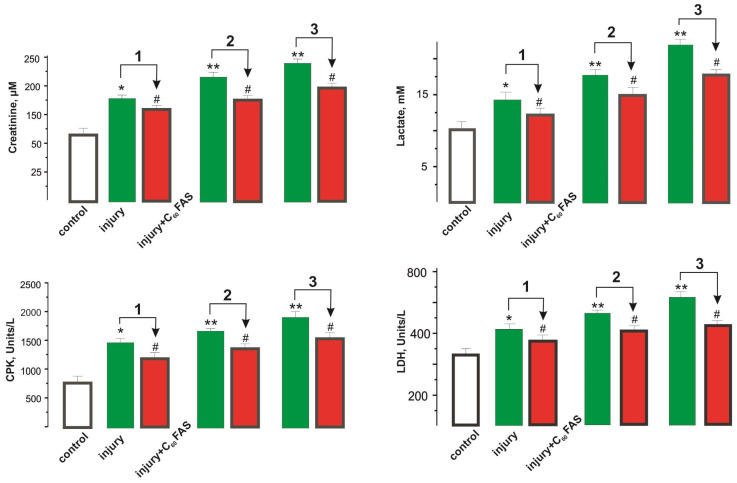
The biochemical indices of fatigue processes’ development (the levels of creatinine and lactate and the activities of CPK and LDH in blood plasma) 15 days after soleus muscle injury: injury and injury + C_60_FAS—the injury group (*n* = 30) and the injury group against the background of C_60_FAS injections (*n* = 30), respectively; 1, 2, and 3—degrees of severity of muscle injury; * *p* < 0.05 relative to the control group (*n* = 30); ** *p* < 0.04 relative to the control group (*n* = 30); # *p* < 0.05 relative to the injury group (*n* = 30).

Figure 5 shows that the creatinine concentration increased from 54 ± 1 µM in the control group to 177 ± 7, 224 ± 6, and 241 ± 8 µM at injury severity grades 1, 2, and 3, respectively. C_60_FAS injection resulted in its reduction by 11 ± 1%, 25 ± 1%, and 33 ± 2% at the 1st, 2nd, and 3rd injury severity levels, respectively, relative to the injury group. The decrease in the creatinine fraction, in our opinion, was caused by the C_60_ fullerene protective effect at the early stage of the pathological process by reducing inflammatory reactions and the ability to protect myocyte membranes from nonspecific free-radical destruction by absorption of reactive oxygen species (ROS) [18].

During the development of inflammatory reactions after the initiation of muscle injury, a significant depletion of cellular energy substances, especially ATP (adenosine triphosphate), occurs, which leads to a sharp disturbance in homeostasis and loss of ionic gradient across cell membranes. This causes the accumulation of lactate and H^+^ ions and, consequently, the acidification of the pH of intra- and extracellular environments [31]. Thus, ionic changes impair the muscle’s ability to respond to electrical impulses, hinder the development of excitation, and lead to a decrease in muscle strength. Consequently, the lactate level is an important marker for assessing the performance of the injured muscle.

The analysis of blood lactate content showed an increase from 10 ± 1 mM in the control group to 15 ± 1, 17 ± 1, and 20 ± 2 mM at the 1st, 2nd, and 3rd injury severity levels, respectively. The C_60_FAS injection reduced this index by 12 ± 1%, 19 ± 1%, and 37 ± 2% at the 1st, 2nd, and 3rd injury severity levels, respectively, relative to the trauma group (Figure 5).

One of the known markers of muscle fatigue is a change in the activity of CPK, an enzyme from the energy supply system of skeletal muscle cells. During intensive muscle functioning, this enzyme is released from the cells and, accordingly, creates an increase in CPK activity in the blood [30].

The increase in CPK activity from 620 ± 17 Units/L in the control group to 1410 ± 23, 1620 ± 32, and 1840 ± 29 Units/L at the 1st, 2nd, and 3rd degrees of injury severity, respectively, indicates an increased energy load on the injured muscle due to increased connective tissue and the dysfunction of post-traumatic muscle fibers [32]. CPK activity decreased by 24–28 ± 2% relative to the trauma group when injected with C_60_FAS (Figure 5).

The change in the activity of LDH, an enzyme that catalyzes the oxidation of lactic acid, allowed us to assess the performance of the injured muscle after its prolonged stimulation [31].

The increase of LDH activity from 310 ± 9 Units/L to 390 ± 13, 413 ± 15, and 493 ± 14 Units/L at the 1st, 2nd, and 3rd degrees of injury severity, respectively, indicates the development of significant dysfunctions of the neuromuscular apparatus and, as a consequence, fatigue processes. The C_60_FAS injection decreased LDH activity by 14 ± 1%, 21 ± 1%, and 37 ± 2% at the 1st, 2nd, and 3rd injury severity levels, respectively, relative to the injury group (Figure 5).

Summarizing the above biochemical results, we can conclude that the administration of C_60_FAS in a daily dose of 1 mg/kg into the injured soleus muscle during the experiment improved the values of the investigated biochemical indices of the animals’ blood by 15–34 ± 2% relative to the trauma group. This can be explained as follows: C_60_ fullerenes, as powerful antioxidants, can effectively inactivate ROS [18,19,20], protecting myocytes from damage and reducing the inflammation of the injured muscle, thereby reducing fibrosis [33] and thus preventing the development of post-traumatic soleus muscle dysfunction, which requires further preclinical testing.

## 3. Materials and Methods

### 3.1. Preparation of C_60_FAS

C_60_FAS was prepared according to the method [34]. Briefly, we used a saturated solution of C_60_ fullerene (purity > 99.99%) in toluene with a C_60_ molecule concentration corresponding to maximum solubility near 2.9 mg/mL and the same amount of distilled water in an open beaker. The two phases formed were treated in an ultrasonic bath (8 Hz, 8 h). The procedure was continued until the toluene had completely evaporated and the water phase became yellow-colored. Filtration of the aqueous solution (the pore size of the filter was smaller than 2 mm) allowed the separation of the product from undissolved C_60_ fullerene. The resulting C_60_FAS, with a maximum concentration of 0.15 mg/mL, is a typical colloid containing both single C_60_ molecules (~0.7 nm) and their nanoaggregates up to ~100 nm in size, and its zeta potential value was −25 ± 2 mV [35]. Moreover, C_60_FAS was stable for 12–18 months at a storage temperature of +4–20 °C.

### 3.2. In Vivo Experiments

The experiments were performed on male Wistar rats aged 1 to 1.5 months (at the end of the experiment). The rats were kept under controlled environmental conditions (21 °C, 12 h light–12 h dark cycle) with free access to water and standard rodent chow. The study protocol (No. 9, dated 4 September 2023) was approved by the Bioethics Committee of the ESC “Institute of Biology and Medicine” of Taras Shevchenko National University of Kyiv in accordance with the rules of the European Convention for the Protection of Vertebrate Animals Used for Experimental and Other Scientific Purposes and the norms of biomedical ethics in accordance with the Law of Ukraine No. 3447-IV of 21.02.2006, Kyiv, “On the Protection of Animals from Cruelty” during biomedical research.

Muscle injury was induced by compressing the soleus muscle for 1 (the 1st degree of injury severity), 2 (the 2nd degree of injury severity), and 3 (the 3rd degree of injury severity) min with a clamp under a pressure of 3.5 kg/cm^2^ [36,37]: We made small incisions in the skin of laboratory animals, where the branches of the crusher device were placed to prevent mechanical impact on the nearby gastrocnemius muscle. This process was controlled visually using a FIM-17 fiber optic microscope (Grandway Telecom Tech., Shanghai, China).

The applied crush syndrome leads to the destruction of myocytes, specifically the release of their components (creatine kinase, lactic acid, and myoglobin) into the extracellular environment, which serves as a marker of the severity of muscle injury. The 1st degree of severity of muscle injury is characterized by the manifestation of numerous local intramuscular hematomas and ruptures of individual muscle cells without damage to the integrity of the muscle membranes; a characteristic manifestation of the 2nd degree of severity of muscle injury is ruptures of the fascial structures of the muscle, damage to the muscle innervation, inflammation and swelling of the muscle, damage to blood vessels, and, as a result, large superficial and intramuscular hematomas; the 3rd degree of severity of muscle injury is characterized by ruptures of muscle sheaths, fascia and subfascial structures, muscle tendons, blood vessels, nerves, auxiliary apparatus of the muscle, and tissues adjacent to the muscle [36,37,38,39].

Experimental animals were divided into the following groups: control (*n* = 30), model injury of the 1st, 2nd, and 3rd severity degrees (*n* = 30), and model injury of the 1st, 2nd, and 3rd severity after administration of C_60_FAS (*n* = 30). C_60_FAS was administered intraperitoneally daily throughout the experiment, starting on the first day immediately after the initiation of muscle injury.

Previously we investigated the impact of different doses of C_60_FAS (0.5, 1, 1.5, and 2 mg/kg) on various in vivo models of muscle pathologies [22,23,24,25] and found that the 1 mg/kg of C_60_FAS dose demonstrated the highest efficacy in the therapy. Therefore, this dose was chosen for these experiments. In addition, it is significantly lower than the LD_50_ value (lethal dose, 50%), which was 600 mg/kg in the case of oral administration to rats [18] and 721 mg/kg in the case of intraperitoneal administration to mice [35]. It is also important to note that after intravenous administration to mice, C_60_ fullerenes accumulate predominantly in the blood, spleen, stomach, and liver and are excreted from the body within 72 h, mainly with urine [40].

All experimental studies were conducted during a 15-day post-injury period. This is due to the fact that the main processes of repairing damaged muscles in the conditions of their natural post-traumatic recovery last for 12–15 days [41].

### 3.3. Materials for Biomechanical Analysis

Animals were anesthetized (to study the functional activity of the soleus muscle) by intraperitoneal injection of nembutal (40 mg/kg). The soleus muscle of the rat was freed from the surrounding tissues. Its tendon part was cut across the distal part of the lumbar spine. For modulated stimulation of efferents in the L7-S1 segments, the ventral roots were cut at the sites of their exit from the spinal cord. Electrical pulses performed a stimulation of soleus muscle efferents with a duration of 2 ms, generated by a pulse generator through platinum electrodes. The current strength at which the muscle started to contract was considered the threshold, and further stimulation was performed at a strength of 1.3–1.4 of that threshold. The external load on the soleus muscle was controlled using a system of mechanical stimulators. To record the force of skeletal muscle contraction, an original strain gauge setup consisting of force and length sensors, a synchronous pulse generator, and a temperature control system was used [22,23,24,25].

When analyzing the contractile activity of the soleus muscle, the following basic biomechanical parameters were evaluated [22,23,24,25]:

- integrated muscle power—calculated area (S) under the power curve using Origin 9.4 software, which is an indicator of the overall performance of the muscle at applied stimulation pools;
- F_min_ and F_max_—minimum and maximum forces of contraction;
- t_0_—the time between the end of stimulation and the force curve reaching the initial level;
- t_start_—the time between the beginning of stimulation and the beginning of muscle contraction;
- t_50_—time of contraction force reduction by 50% of the initial level.

### 3.4. Materials for Biochemical Analysis

Blood biochemical indices of experimental animals, such as creatinine and lactate levels and activities of CPK and LDH, which are the most commonly used clinical markers of muscle injury [42], were determined using the following diagnostic equipment: biochemical analyzers RNL-200 and JN-1101-TR2 (Amsterdam, The Netherlands).

### 3.5. Statistical Analysis

The statistical evaluation of the experimental results was performed using the procedure of variance analysis (ANOVA) with mixed design. Two between-group factors were supposed: (1) injury (three levels—the 1st, 2nd, and 3rd degrees of severity); (2) C_60_FAS treatment (two levels—no and use of C_60_FAS). The Shapiro–Wilk *W*-test was used to test for normality. Levene’s test was used to assess the equality of variances across groups. Multiple pairwise comparisons between different groups and conditions were performed by the Bonferroni post-hoc test. The differences between the groups were considered significant at *p* < 0.05. Each of the experimental force curves was the result of averaging 10 similar tests. Each biochemical measurement was carried out at least three times. The statistical evaluation was performed using the software package Statistica 8.0 (Dell Technologies Inc., Round Rock, TX, USA).

## 4. Conclusions

To date, the powerful antioxidant properties of C_60_ fullerenes have been widely used in biomedicine. Due to the negative effects of ROS in oxidative stress processes, antioxidants are required to protect injured muscles. Here, the effect of water-soluble C_60_ fullerenes (daily intramuscular dose of 1 mg/kg) on the process of the restoration of contractile activity of rat skeletal muscle on the 15th day after the initiation of injury of varying severity was studied for the first time. The revealed improvement in the dynamics of *the* soleus muscle functioning by 28–40 ± 2% and biochemical indices of rat blood by 15–34 ± 2% opens the prospect of applying C_60_ fullerenes as potential nanoagents that are able to effectively correct the pathological state of skeletal muscle arising from its mechanical trauma.

## Data Availability

The original contributions presented in the study are included in the article; further inquiries can be directed to the corresponding author.
